# Over-expression of miR-183-5p or miR-492 triggers invasion and proliferation and loss of polarity in non-neoplastic breast epithelium

**DOI:** 10.1038/s41598-022-25663-8

**Published:** 2022-12-20

**Authors:** Nataly Naser Al Deen, Nadia Atallah Lanman, Shirisha Chittiboyina, Sabreen Fostok, Rihab Nasr, Sophie Lelièvre, Rabih Talhouk

**Affiliations:** 1 Department of Biology, Faculty of Arts and Sciences, American University of Beirut, P.O. Box: 11-0236, Beirut, Lebanon; 2 Purdue University Center for Cancer Research, Purdue University, Indiana, USA; 3 Department of Comparative Pathobiology, Purdue University, Indiana, USA; 4 Department of Basic Medical Sciences, College of Veterinary Medicine, Purdue University, Indiana, USA; 5 Centers for Disease Control and Prevention, CDC, National Institute for Occupational Safety and Health, Cincinnati, USA; 6 Department of Anatomy, Cell Biology and Physiological Sciences, Faculty of Medicine, American University of Beirut, Beirut, Lebanon; 7 Institut de Cancérologie de L’Ouest (ICO), Angers, France

**Keywords:** Breast cancer, Cancer genomics, Tumour biomarkers, Cancer, Cell biology, Cell polarity

## Abstract

microRNAs (miRNAs) serve as novel noninvasive cancer biomarkers. In an HMT-3522 S1 (S1) breast epithelial risk-progression three-dimensional (3D) culture model, non-neoplastic S1 cells form a fully polarized epithelium. When silenced for the gap junction and tumor suppressor Cx43, Cx43-KO-S1 cells recapitulate pre-neoplastic phenotypes observed in tissues at risk for breast cancer in vivo. To delineate the role of miRNAs in breast tumorigenesis and identify key miRNA players in breast epithelial polarity, the miRNA profile specific to Cx43 loss in Cx43-KO-S1 compared to S1 cells was sequenced, revealing 65 differentially expressed miRNAs. A comparative analysis was conducted between these miRNAs and tumor-associated miRNAs from a young Lebanese patient validation cohort. miR-183-5p, downstream of Cx43 loss, was commonly upregulated in the patient cohort and the 3D culture model. miR-492, not attributed to Cx43 loss, was only specifically up-regulated in the young Lebanese patients. Ectopic expression of either miR-183-5p or miR-492 in S1 cells, through pLenti-III-miR-GPF vectors, resulted in the formation of larger multi-layered acini devoid of lumen, with disrupted epithelial polarity, as shown by an altered localization of Cx43, ß-catenin and Scrib, and decreased nuclear circularity in 3D cultures. Enhanced proliferation and invasion capacity were also observed. Over-expression of miR-183-5p or miR-492, therefore, induces pre-neoplastic phenotypes similar to those reported upon Cx43 loss, and may act as oncomiRs and possible biomarkers of increased breast cancer risk.

## Introduction

Breast cancer is the most common malignancy in Lebanon and the second cause of cancer-related deaths after lung cancer. In 2020, breast cancer accounted for the highest number of new cancer cases (33.7%) in all females across all ages in Lebanon (Globocan 2020^1^), with the highest incidence in the world for women below the age of 40^2^. The incidence of breast cancer in young women is increasing worldwide^2–4^. Despite the availability of therapeutic options, poor survival rate and early onset in women make breast cancer a public health concern. The young Lebanese breast cancer patients in general exhibit low prevalence of deleterious *BRCA* mutations^5^ and present with poor prognosis and aggressive phenotypes due to the lack of diagnostic methods at such an early age^6^.

miRNAs are small (16–29 nucleotides), endogenous, noncoding, single-stranded RNAs that regulate gene expression at the post-transcriptional level and can act as noninvasive cancer biomarkers^7^. Several miRNAs are ubiquitously expressed in different cancer tissue types, while others can act in a tissue-specific, tumor-specific and/or stage-specific manner^8^. miRNAs exert their regulatory functions mostly through down-regulating their target genes, by interacting with the 3′UTRs of coding genes, thus affecting downstream signaling pathways^9^. Through a previous miRNA microarray study performed on Lebanese patients using 45 invasive ductal carcinoma (IDC) and 17 normal adjacent breast tissues, Nassar et al.^10^ reported 74 dysregulated miRNAs. These patients had mostly grade 2 tumors with no distant metastasis, half of which presented with involvement of lymph nodes, and mostly were both estrogen and progesterone receptors positive (ER + and PR +), while quarter had human epidermal growth factor receptor 2 (HER2) over-expression. Of note, almost half of these Lebanese patients were younger than 40 years old, hence, were described as an early-stage breast cancer cohort, and their mRNA microarrays revealed upregulation in cellular growth, proliferation and motility, thus fitting into the more aggressive Luminal B subtype of ER + breast tumors. A cohort with matched stage, histology and metastasis status from The Cancer Genome Atlas (TCGA) dataset was used for comparison with the Lebanese patients miRNome^10^. In order to understand the mechanisms that underlie heightened risk of early breast cancer, preliminary results from the Lebanese young early-stage patient population with aggressive phenotypes were essential for such investigations. We, therefore, recently performed a comprehensive literature review on this young Lebanese cohort’s miRNome, focusing on miRNAs that play role in early events of breast tumorigenesis and contribute to loss of epithelial polarity, and identified 15 tumor-associated miRNAs amongst this cohort that guided our choice of miRNAs to be studied in the 3D culture model^11–16^.

Gap junctions, through mediating gap junction intercellular communication (GJIC), play essential role in the mammary gland development and differentiation, and their lack of organization and communication can lead to tumorigenesis^17^. The GJIC is orchestrated by connexins (Cxs), which are transmembrane proteins that take part in allowing the intercellular exchange between neighboring cells of ions, second messengers and metabolites^18–21^. Our previous and current studies^22–24^ focus on Cx43, which plays fundamental roles during mammary gland development^25,26^ and differentiation^27^. Cx43 has been reported as a tumor suppressor^23,24,28^. When Cx43 is lost and/or mislocalized, it contributes to breast cancer initiation^29^ and progression^30^ and places women with obesity at an increased risk of acquiring breast cancer^31,32^. Breast cancer patients with loss in Cx43 expression present with poor prognosis, increased metastasis and poor survival rates^28^. S1 cells represent a non-neoplastic luminal human breast epithelial 3D culture line, with apically localized Cx43 in growth-arrested and basoapically polarized acini with a central lumen. Hence, S1 cells recapitulate normal human breast tissue architecture^29^. Silencing Cx43 expression in S1 cells via Cx43-shRNA (Cx43-KO-S1) resulted in perturbed apical polarity, mitotic spindle misorientation and cell multilayering without creation of a lumen^29^. This phenomenon was accompanied by enhanced cell cycle entry, motility, and invasion^29,30^. The observed phenotypic changes in Cx43-KO-S1 cells in 3D cultures recapitulate premalignant phenotypes previously seen in mouse models with lesions in ductal hyperplasia^33^. Cx43-KO-S1 cells, thus, serve as a precancerous culture model while S1 cells serve as a non-neoplastic model. Therefore, to understand the potential roles that the tumor-associated miRNAs from the young Lebanese cohort might play in breast cancer initiation, this risk-progression 3D culture model was utilized; to investigate whether patient-associated miRNAs are capable of inducing, in S1 cells, pre-neoplastic phenotypic changes similar to ones observed in Cx43-KO-S1 cells.

miRNA-Sequencing (miRNA-Seq) analysis was therefore performed on Cx43-KO-S1 cells compared to S1 cells, and revealed 65 differentially expressed miRNAs attributed to Cx43 loss. A comparative analysis of these miRNAs relative to tumor-associated miRNAs from the young Lebanese breast cancer patient cohort^11–16^ was performed to delineate the role of miRNAs in breast tumorigenesis and to identify key miRNA players in breast epithelial polarity^29,30^. miR-183-5p (downstream of Cx43 loss and commonly upregulated in the patient cohort and in Cx43-KO-S1 cells) and miR-492 (not attributed to Cx43 loss, and only specific to the young Lebanese patient cohort) were chosen here for stable over-expression in the non-neoplastic S1 cells. This study was able to reveal a shift from a normally polarized to a precancerous phenotype in 3D culture upon the over-expression of miR-183-5p or miR-492 through disrupting the breast epithelial polarity, similar to what we reported previously upon Cx43 loss^29,30^.

## Results

### Selection of miR-183-5p and miR-492through a comparative analysis from miRNomes of a breast epithelial 3D risk-progression culture model and a young early-stage patient cohort

**Figure 1:**
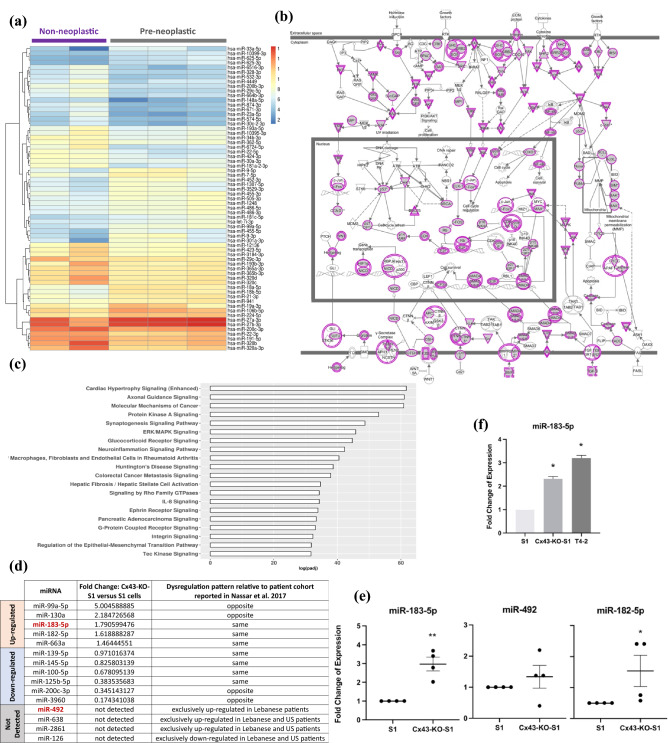
Identification and validation of candidate tumor-associated miRNAs in a breast cancer risk-progression 3D culture model. (**a**) A heatmap revealing the expression patterns of the 65 significant differentially expressed miRNAs attributed to Cx43 loss in Cx43-KO-S1 cells compared to S1 cells cultured in 3D conditions (fold change > 2). Orange depicts up-regulated miRNAs (29 miRNAs), and blue depicts down-regulated ones (36 miRNAs) in pre-neoplastic Cx43-KO-S1 cells compared to non-neoplastic S1 counterparts. The dendrogram depicts hierarchical clustering of miRNAs based on expression patterns. (**b**) Ingenuity Pathway Analysis (IPA) revealed molecular mechanisms of cancer canonical pathways as the most significantly enriched pathway in Cx43-KO-S1 cells compared to S1 cells. Colored nodes indicate mRNA targets of miRNAs that were significantly differentially expressed in the comparison. (**c**) Functional enrichment analysis performed in IPA of the predicted target mRNAs (by TargetScan^36^) for the top 20 enriched pathways for all dysregulated miRNAs in Cx43-KO-S1 cells compared to S1 cells revealed mainly pathways involved in cancers. The y-axis shows the enriched canonical pathways, and the x-axis shows the log_10_ adjusted p-value, which was adjusted for multiple comparisons using the Benjamini–Hochberg method. (**d**) A table showing the regulation pattern of miRNAs from miRNA-Seq of the breast cancer risk-progression 3D culture model as compared to tumor-associated miRNAs from young Lebanese patient cohort with early-stage breast cancer (reported by Nassar et al., 2017) that are involved in early events of breast tumorigenesis and affect epithelial polarity (from Nasser Al Deen et al., 2021). Orange highlight panel refers to up-regulated miRNAs and blue highlight to down-regulated miRNAs in Cx43-KO-S1 compared to S1 cells. Grey highlights refer to the patient tumor-associated miRNAs involved in epithelial polarity that were not detected in the cells’ miRNome. (**e**) RT-qPCR validation (n = 4) in 3D cultures of Cx43-KO-S1 compared to S1 cells for candidate miRNAs using RNU6B as an endogenous control. (**f**) RT-qPCR validation of miR-183-5p in the breast epithelial risk progression culture model in 2D cultures showing that miR-183-5p expression increases with the increased progression of the series. ** denotes a p-value < 0.01, and * denotes a p-value < 0.05 based on unpaired t-test with Welch’s correction.

miRNA sequencing revealed 29 significantly up-regulated (44.6%) and 36 significantly down-regulated (55.4%) mature miRNAs in response to Cx43 silencing in Cx43-KO-S1 cells compared to S1 cells, as depicted by the heatmap (Fold Change > 2 and adjusted p-value < 0.05) (Fig. 1a). Of the 65 differentially expressed miRNAs in Cx43-KO-S1 compared to S1 cells, 38 miRNAs (60%) were significantly involved in cancer-related pathways, including the ones tabulated in Fig. 1d. Using experimentally validated results from Ingenuity Pathway Analysis (IPA), molecular mechanisms of cancer canonical pathways were the most significantly enriched pathways in the miRNAs that were dysregulated in Cx43-KO-S1 cells compared to S1 cells (Fig. 1b). Furthermore, functional enrichment analysis performed in IPA of predicted target mRNAs by TargetScan^36^ for the top 20 enriched pathways for all dysregulated miRNAs in Cx43-KO-S1 cells compared to S1 cells revealed that Protein Kinase A signaling, ERK/MAPK signaling, signaling by Rho GTPases, inflammation by IL-8 signaling and regulation of epithelial to mesenchymal transition (EMT), hence well-known molecular mechanisms of cancer, were among the most enriched pathways in the differentially expressed miRNAs upon Cx43 loss (Fig. 1c). We then performed a comparative analysis of these dysregulated miRNAs from the breast epithelial risk progression culture model and tumor-associated miRNAs from a young Lebanese patient cohort with early-stage breast cancer^10^, in order to select candidate miRNAs that might participate in the pre-neoplastic phenotypes^29,30^.

A previous literature review by our group showed that fifteen tumor-associated miRNAs from the patient cohort have been reported in epithelial polarity and cancer-related pathways^16^. The corresponding dysregulation pattern of these cancer-associated miRNAs in the risk-progression cell culture miRNome was tabulated (Fig. 1d). Only miRNAs that were up-regulated in patient tissues compared to normal adjacent tissues and/or in pre-neoplastic Cx43-KO-S1 cells as compared to non-neoplastic S1 cells were filtered in Fig. 1d. The first selection criteria for choosing a candidate miRNA for over-expression in the S1 cells was to look into commonly up-regulated miRNAs in the young patient population and upon Cx43 loss in the cell lines. Therefore, miR-183-5p, miR-182-5p, and miR-663a were explored, and the highest expression signature was considered. The most upregulated common miRNA in breast cancer patient samples (fold change = 10) and Cx43-KO-S1 cells (fold change = 1.79) was miR-183-5p, followed by miR-182-5p (fold change = 5.96 in patients and 1.62 in cells), followed by miR-663a (fold change = 1.56 in patients and 1.46 in cells) (Fig. 1d),^16^. miR-182 belongs to the miR-183 family of miRNAs^35^, therefore, miR-183-5p was chosen as the first candidate miRNA. RT-qPCR validation confirmed the up-regulation of miR-183-5p in Cx43-KO-S1 cells cultured in 3D conditions (fold change = 3) as compared to S1 cells using RNU6B as an endogenous control (Fig. 1e). Additionally, RT-qPCR validation in 2D cultures of the risk-progression series, using RNU6B as an endogenous control, showed that miR-183-5p was significantly up-regulated by 2.5 folds in Cx43-KO-S1 cells and further up-regulated by 3.5 folds in the invasive ductal carcinoma T4-2 cells^34^ as compared to their non-neoplastic counterpart, the S1 cells (Fig. 1f). Therefore, miR-183-5p was chosen for over-expression in the non-neoplastic S1 cells. RT-qPCR analyses additionally showed that miR-182-5p, belonging to the miR-183 family cluster^35^, was also significantly up-regulated (2.5 folds) in Cx43-KO-S1 compared to S1 cells, using RNU6B as an endogenous control (Fig. 1e).

Next, to explore which miRNA pathways in addition to those under Cx43 control may be associated with heightened breast cancer risk, we also focused on the sub-set of miRNAs that were not detected in the sequencing results of the cultured epithelia but were only up-regulated in the patient miRNome (Fig. 1d, grey panel). Only miR-492 was not detected in the cells’ miRNome and was exclusively up-regulated in the Lebanese early-stage validation cohort but not in the matched TCGA patient cohort of young women. Therefore, miR-492 appears to be specific to the Lebanese population with the highest known incidence in young population worldwide^2,10^. An alarmingly high percentage of Lebanese women are diagnosed under the age of 40 (22% of cases compared to 6% in Western populations), with a mean age at diagnosis 10 years younger than in Western countries^2^. RT-qPCR further confirmed that miR-492 was not significantly dysregulated in Cx43-KO-S1 cells as compared to control S1 cells in 3D cultures using RNU6B as an endogenous control (Fig. 1e). Thus, miR-492 (specific to the young Lebanese cohort miRNome) was chosen together with miR-183-5p for over-expression in the non-neoplastic S1 cells.

### Over-expression of miR-183-5p and miR-492 in S1 cells disrupts the epithelial architecture associated with normalcy

**Figure 2:**
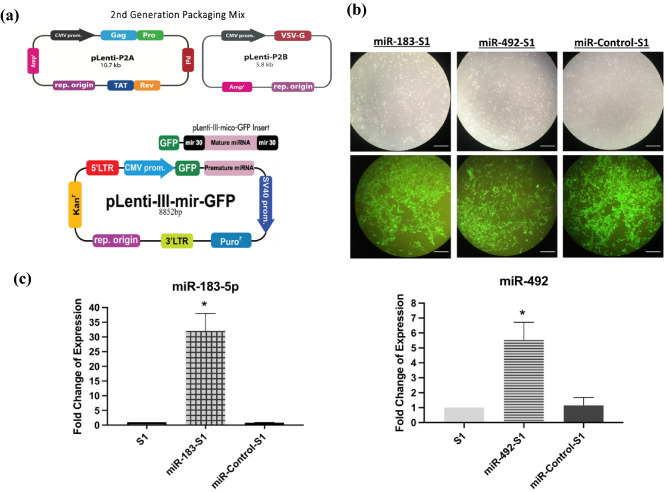
Stable over-expression of miR-183-5p and miR-492 in non-neoplastic S1 cells using pLenti-III-miR-GPF tagged vectors. (**a**) The maps specific to pLenti-III-miR-GPF tagged vectors as well as the 2nd generation packaging mix used. (**b**) Microscopy images showing pLenti-III-miR-GFP viral infection in both miR-183-S1 and miR-492-S1 infected cells on day 25 in culture (16 days post-puromycin treatment at 0.5 ug/ul). The top panel shows phase contrast images while the bottom one shows fluorescence images. pLenti-miR-control (empty vector), miR-Control-S1, was used as a control. (**c**) RT-qPCR quantification showed that over-expressing miR-183-5p and miR-492 in S1 cells significantly upregulated the expression of miR-183 by 32 folds and miR-492 by 6 folds, respectively. This was done in triplicates as compared to uninfected S1 controls and miR-Control-S1 cells in 3D cultures using RNU6B as an endogenous control. * denotes a p-value < 0.05 using one-tailed unpaired T-test.

The pLenti-III-miR-GPF tagged plasmids were used to generate stable vectors of the mature miR-183-5p and miR-492 miRNAs. The pLenti-miR-control (empty vector) was used as a control. A 2^nd^ generation packaging mix was used with the amphoteric 293 T cells to package the viruses. Infection and selection of plenti-miR-183-5p, plenti-miR-492 and plenti-miR-Control in S1 cells were assessed through fluorescence microscopy (Fig. 2a,b). RT-qPCR quantification confirmed upregulation of the expression of miR-183-5p by an average of 32-fold and of miR-492 by an average of sixfold in three separate replicates as compared to un-infected S1 controls and pLenti-miR-control infected S1 cells in 3D cultures using RNU6B as an endogenous control (Fig. 2c).

### Over-expressing miR-183-5p or miR-492 resulted in larger multicellular structure, loss of lumen formation, and disruption in localization of Cx43, β-catenin and Scrib

**Figure 3:**
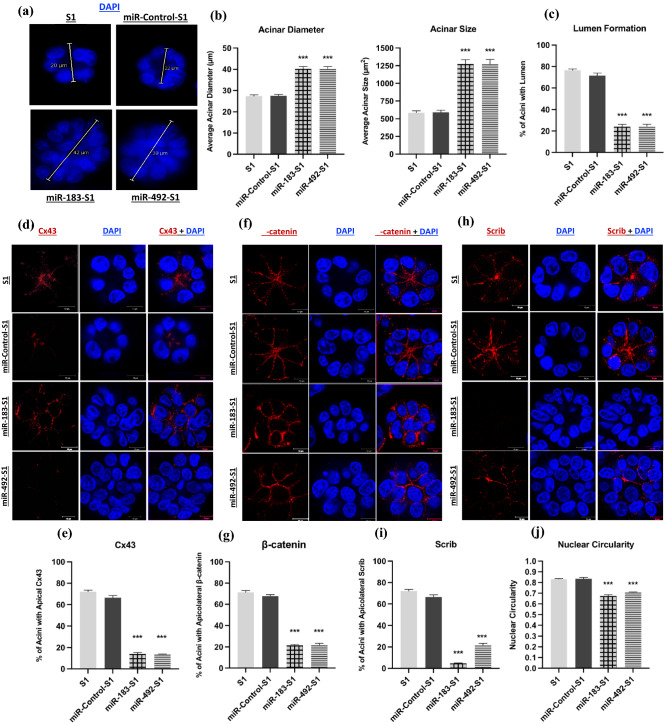
Immunofluorescence staining and quantification characterizing phenotypic changes observed in non-neoplastic S1 cells upon miR-183 and miR-492 over-expression. (**a**) Representative fluorescence images of S1, miR-Control-S1, miR-183-S1 and miR-492-S1 acini showing their diameter. Nuclei were counterstained with DAPI (blue). (**b**) Bar graphs showing acinar diameter (µm) and size, reported as acinar area (µm^2^) and (**c**) scoring for the presence/absence of lumen. Size measurements and scoring were done on at least 100 acini across 12 regions. (**d**) Localization of Cx43 (red) with nuclei counterstained with DAPI (blue) and (**e**) scoring of acini with apical Cx43 distribution, reported as percentages. (**f**) Localization of β-catenin (red) with nuclei counterstained with Dapi (blue) and (**g**) scoring of acini with apico-lateral β-catenin distribution, reported as percentages. (**h**) Localization of Scrib (red) with nuclei counterstained with DAPI (blue) and (**i**) scoring of acini with apico-lateral Scrib distribution, reported as percentages and (**j**) scoring of acini for nuclear circularity. *** denotes a p-value < 0.001, ** denotes a p-value < 0.01 and * denotes a p-value < 0.05 by unpaired *t*-test.

Over-expressing miR-183-5p or miR-492 resulted in larger multicellular structure in 3D culture with an average size of ≈ 1275 µm^2^ (± 62 µm^2^) and average diameter of ≈ 40 µm (± 1 µm)) as compared to the average size of ≈ 590 µm^2^ (± 30 µm^2^) and average diameter of ≈ 27 µm (± 0.7 µm) in both S1 and miR-Control-S1acini (Fig. 3a,b). The larger acini obtained from miR-183- and miR-492-overexpressing cells were also devoid of lumen formation (in 76% of the multicellular structures) and exhibited cell multi-layering, compared to miR-Control-S1 and noninfected S1 acini in which only 28.5 and 24% of acini did not show a lumen, respectively (Fig. 3a,c). Immunostaining also revealed an alteration in the apical localization of Cx43 in both miR-183-S1 and miR-492-S1 multicellular structures as compared to the phenotypically normal S1 acini, (14% of the acini exhibiting apico-lateral distribution of Cx43 in miR overexpressing structures compared to 70% in S1 and miR-Control-S1 acini (Fig. 3d,e). The apico-lateral distribution of the adherens junction component β-catenin was only found in 20% of the acini compared to 70% in S1 and miR-Control-S1 acini (Fig. 3f,g). These results were similar to the ones that we previously reported in the Cx43-KO-S1 structures upon knock-down of Cx43 through shRNA^29,30^. The apico-lateral distribution of Scrib, a regulator of apical polarity, was altered in 80% of miR-492-S1 multicellular structures, and in 95% in miR-183-S1 structures as compared to miR-control S1 acini in which Scrib distribution is altered in only 30% of the structures (Fig. 3h,i). Furthermore, nuclear circularity measured based on DAPI was significantly lower in miR-183-S1 nuclei ≈ 0.7 and in miR-492-S1 nuclei ≈ 0.68 as compared to ≈ 0.83 in S1 and miR-Control-S1 nuclei in 3D cultures (Fig. 3j). With the decrease of nuclear circularity being hallmark of cancerous development, these results reinforce the loss of tissue homeostasis represented by epithelial polarity disruption that is already associated with conditions of increased breast cancer risk^29,37^.

### Over-expressing miR-183-5p and miR-492 in non-neoplastic S1 cells resulted in phenotypes associated with cancer development and adverse prognosis

**Figure 4:**
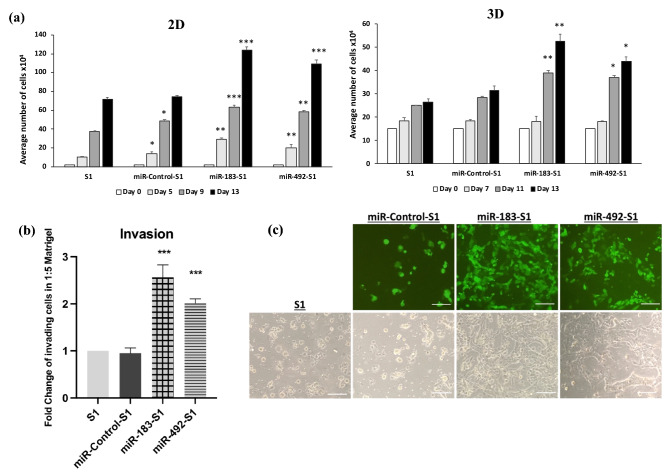
Assessment of proliferation and invasion upon over-expressing miR-183 and miR-492 in non-neoplastic S1 cells. (**a**) S1, miR-Control-S1, miR-492-S1 and miR-183-S1 cells were cultured in 2D or 3D conditions. Proliferation rate was assessed by cell counting on days 5, 9 and 13 in 2D cultures and on days 7, 11 and 13 in 3D cultures. Experiments were repeated at least three times. The values depicted in the histograms are the means (± standard error of the mean, SEM) of cell counts. (**b**,**c**) Invasion of S1, miR-Control-S1, miR-492-S1 and miR-183-S1 cells cultured in diluted Matrigel (1:5) for 72 h. Invasion was assessed by trans-well cell invasion assay. The values depicted in the histogram (**b**) denote the fold change of the number of invading cells (± SEM) from three separate experiments. (**c**) Representative images of the invading cells after 72 h of being cultured in diluted Matrigel (1:5). Scale bar represents 80 pixels at 10X magnification. *** denotes a p-value < 0.001, ** denotes a p-value < 0.01 and * denotes a p-value < 0.05 by unpaired *t*-test.

The 2D cultures revealed a significant increase in cell counts, by 188%, 69% and 73% in the miR-183-S1 population and by 102%, 56.5% and 52% in the miR-492-S1 population on days 5, 9 and 13, compared to the control S1 cells. In contrast, miR-Control-S1 cells only revealed a 37% and 30% significant increase in cell counts on days 5 and 9, respectively, but by day 13 (after cells become quiescent), there was no significant difference in cell count between miR-Control-S1 cells and S1 cells (Fig. 4a left panel). Similarly, the 3D cultures of miR-183-S1 cells revealed a significant increase in cell counts, by 56% and 98% and that of miR-492-S1 by 48% and 66% on days 11 and 13, respectively, compared to control S1 cells. miR-Control-S1 cells cultured under 3D conditions did not reveal any significant increase in cell counts on neither of the days compared to S1 cells (Fig. 4a right panel). The number of dead cells was negligible and did not differ between the two groups at the different time points (data not shown). In addition, in transwell cell invasion assay, miR-183-S1 cells showed a significant increase of 2.56 folds and miR-492-S1 cells of 2 folds in the number of Matrigel-invading cells compared to control S1 cells. miR-Control-S1 cells did not show any significant increase in invading cells compared to S1 cells (Fig. 4b,c).

**Figure 5:**
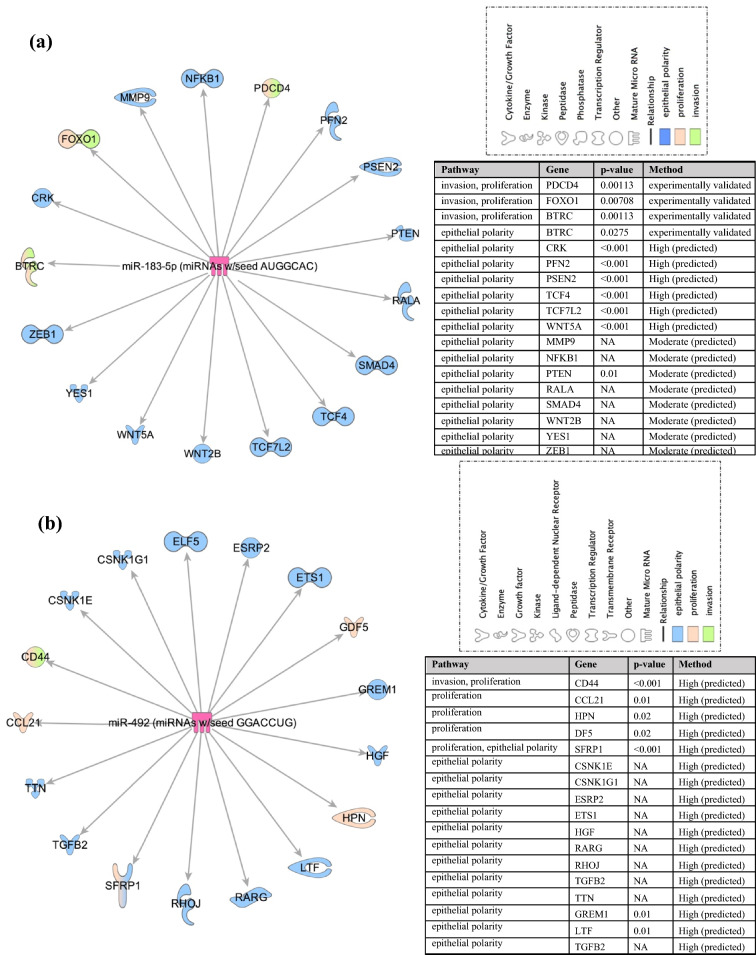
Potential downstream targets of miR-183 and miR-492 involved in pre-tumorigenic pathways. IPA was used to plot the predicted mRNAs (with high and moderate confidence) and experimentally validated mRNA targets downstream of (**a**) miR-183-5p and (**b**) miR-492 that are involved in pre-tumorigenic pathways like epithelial polarity, invasion and proliferation pathways in breast cell lines and tissue biopsies.

**Figure 6:**
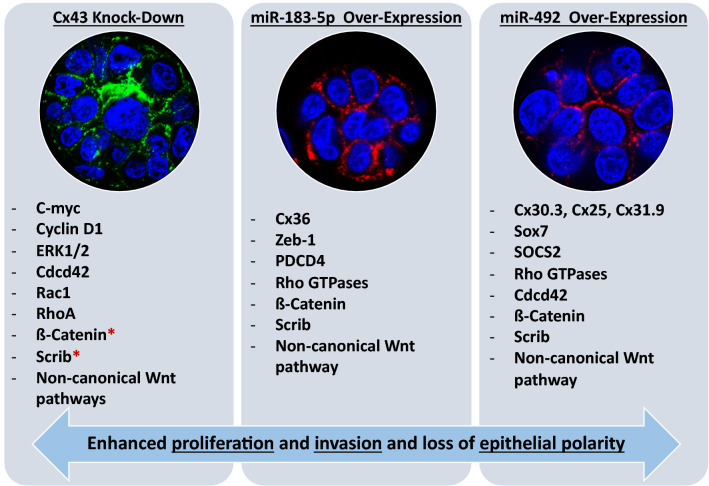
Comparative summary of validated and predicted players in increasing proliferation, invasion and disrupting epithelial polarity upon the loss of Cx43, over-expression of miR-183-5p or over-expression of miR-492. The first panel represents major players/pathways that drive the pretumorigenic phenotypes observed in S1 cells upon Cx43 loss, as reported by Fostok et al.^30^ and Bazzoun et al.^29^. All proteins were up-regulated upon Cx43 loss. The second panel represents major players/pathways that are predicted to drive the pretumorigenic phenotypes observed inS1 cells upon over-expression of miR-183. All genes were predicted to be down-regulated. The third panel represents major players/pathways that drive the pretumorigenic phenotypes observed in S1 cells upon over-expressing miR-492. All reported genes were predicted to be down-regulated. * denotes that total levels were unaffected but their localization along the cell was altered. Each cell line is illustrated by a representative acinar aggregate devoid of lumen formation and with mislocalization of β-catenin (green in the left panel and red in the rest) from apicolateral membrane domains in glandular structures or acini formed in 3D culture, suggesting the loss of apical polarity. Nuclei were counterstained with DAPI (blue).

To identify potential downstream targets of miR-183 and miR-492 that might be driving pre-neoplastic phenotypes, IPA was used. Experimentally validated and predicted (with high or moderate confidence) mRNA targets downstream of miR-183 (Fig. 5a) and miR-492 (Fig. 5b) that are implicated in invasion, proliferation and/or epithelial polarity pathways in breast cells and tissues were illustrated. Specifically, PDCD4, FOXO1 and BTRC downstream of miR-183 are involved in both invasion and proliferation pathways, while the rest of the mRNAs are implicated in epithelial polarity, like MMP9, NFKB1, PTEN, SMAD4, TCF4, WNT2B, WNT5A and ZEB-1(Fig. 5a). Moreover, CD44 was a potential target downstream of miR-492 that is involved in both invasion and proliferation pathways, while CCL21, HPN and GDF5 were targets involved in proliferative pathways. The mRNAs downstream of miR-492 and implicated in epithelial polarity included ETS1, LTF, RARG, RHOJ, TGFB2 and ELF5 (Fig. 5b). In addition, TargetScan was used to predict all downstream targets of miR-183-5p and miR-492, respectively to be compared to the targets reported by Fostok et al.^30^ and Bazzoun et al.^29^ downstream of Cx43 loss, which contributed to the observed pretumorigenic phenotypes. The results showed overlap in pathways and players along Cx43 loss and miR-183-5p and miR-492 over-expression (Fig. 6 and Table 1), however these were not experimentally validated.

**Table 1:**
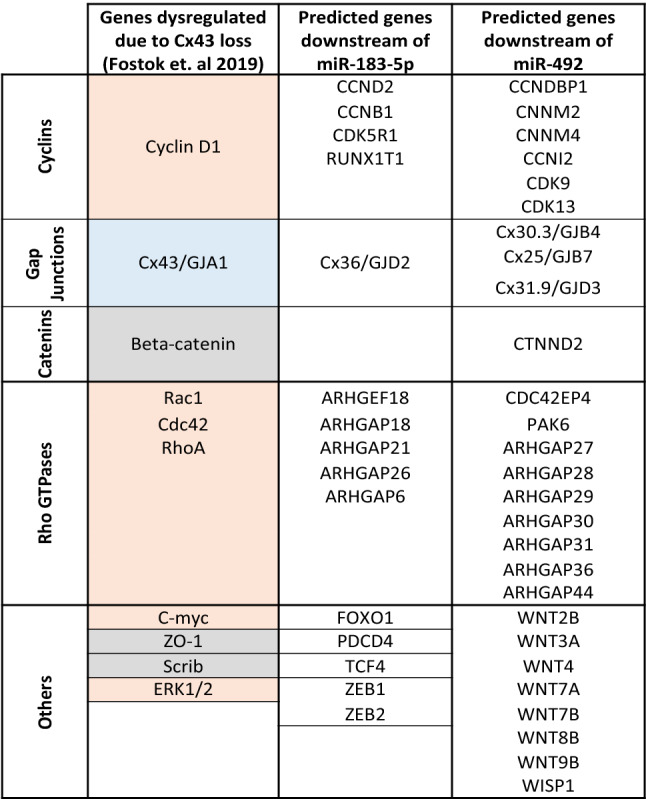
Comparative analysis of targets downstream of Cx43 loss as reported by Fostok et al.^30^ and Bazzoun et al.^29^ that contribute to pretumorigenic phenotypes in Cx43-KO-S1 cells as compared to targets downstream of miR-183-5p and miR-492.

TargetScan was used to predict all downstream targets of miR-183-5p and miR-492, respectively. These targets were extracted and compared to targets reported by Fostok et al.^30^ and Bazzoun et al.^29^ downstream of Cx43 loss, which contributed to the observed pretumorigenic phenotypes. Targets highlighted in orange were found to be up-regulated, in blue were down-regulated while the levels of the ones in grey were unaffected, but their cellular distribution was significantly altered in Cx43-KO-S1 cells compared to S1 cells upon Cx43 loss^29,30^.

**Figure 7:**
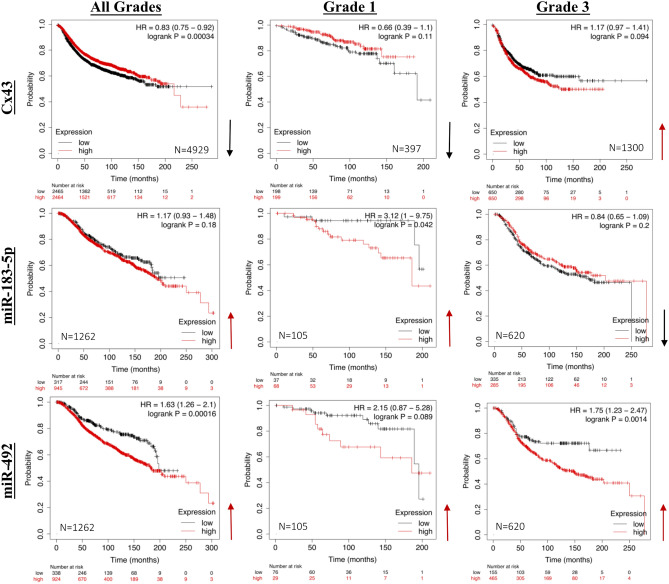
The association of Cx43, miR-183-5p and miR-492 levels with disease prognosis in cohorts of breast cancer patients with low versus high grade tumors. Using all breast cancer mRNA datasets in the Kaplan–Meier Plotter^38,39^, the survival analyses for Cx43 in 4929 patients across all grades, 397 patients with grade 1 and 1,300 patients with grade 3 breast tumors were plotted (upper panel)*.* Using METABRIC breast cancer miRNA dataset in the Kaplan–Meier Plotter the survival analyses for miR-183-5p (middle panel) and miR-492 (lower panel) in 1262 patients across all grades, 105 patients with grade 1, and 620 patients with grade 3 breast tumors were plotted. Red arrows pointing upwards refer to a possible association between poor prognosis in patients and the up-regulation of the respective mRNA/miRNA levels, while a black arrow pointing downwards refers to a possible association between poor prognosis in patients and the down-regulation of the respective mRNA/miRNA levels.

Lastly, to delineate the potential biomarker role of Cx43, miR-183-5p, and miR-492 across different grades of breast tumorigenesis, all breast cancer mRNA datasets in the Kaplan–Meier Plotter were selected^38,39^ and the survival analysis for Cx43 in 4929 patients across all grades, 397 patients with grade 1, and 1300 patients with grade 3 breast tumors revealed that Cx43 seems to significantly associate with poor prognosis when down-regulated across all grades combined and in grade 1 (although the association is not significant) and when up-regulated in high grade (grade 3) breast tumors (Fig. 7, upper panel)*.* Moreover, using METABRIC breast cancer miRNA dataset in the Kaplan–Meier Plotter^38^ the survival analysis for miR-183-5p in 1262 patients across all grades, in 105 patients with grade 1, and 620 patients with grade 3 breast tumors revealed that miR-183-5p when up-regulated significantly associates with poor prognosis in grade 1 breast tumors, however, miR-183-5p seems to associate with poor prognosis (although not significant) when downregulated in grade 3 breast tumors and when up-regulated across all grades (Fig. 7, middle panel). Using the same METABRIC breast cancer miRNA dataset and patient cohort^38^ miR-492 seems to significantly associate with poor prognosis when up-regulated in breast tumors regardless of the tumor grade (Fig. 7, lower panel).

## Discussion

### miR-183-5p and miR-492 over-expression promote pretumorigenic phenotypes in breast epithelial cells, recapitulating phenotypes observed upon Cx43 loss

Cx43 plays essential roles during mammary gland development^25,26^, differentiation^27^ and acts as a tumor suppressor^23,24,28^. Recent research from our group showed that Cx43 loss in Cx43-KO-S1 cells and in archival tissue biopsies was associated with cell multilayering and polarity disruption^29^. Additionally, Cx43-KO-S1 cells exhibited migratory and invasive capacity when cultured under conditions mimicking permissive, or reduced, ECM stiffness^29,30^. S1 non-neoplastic cells grown in 3D culture conditions^40^, in the presence of extracellular matrix basement membrane, represent a model of phenotypically normal and fully polarized breast epithelium^41,42^. This in vitro 3D culture model was, therefore, used to compare the phenotypic effects of the over-expression of candidate tumor-associated miRNAs on the polarized S1 epithelial cells to those observed upon Cx43 loss.

Two breast tumor-associated patient miRNAs were chosen; one downstream of Cx43 loss and another not attributed to Cx43 loss but specific to the young Lebanese patient cohort. miR-183-5p was chosen as it was up-regulated in Cx43-KO-S1 cells, was the most up-regulated miRNA in the early-stage Lebanese breast cancer patient cohort, was up-regulated in matched patients from TCGA^10^, and its up-regulation conferred with the increased risk of cancer progression in the 3D culture model. miR-492, on the other hand, was chosen since it was not detected in the cells’ miRNome and was exclusively up-regulated in the Lebanese early-stage validation cohort, but not in the TCGA patient cohort. Thus, miR-492 seems to be specific to the Lebanese population with the highest known early incidence worldwide^2,10^. While only miR-183-5p was downstream of Cx43 loss, over-expressing miR-183-5p or miR-492 in S1 cells resulted in similar pretumorigenic phenotypic changes that were observed previously upon silencing Cx43 in S1 cells^29,30^. For instance, miR-183-S1 and miR-492-S1 cells exhibited multi-layering and formation of larger acini in 3D cultures devoid of lumen, with disrupted epithelial polarity observed via the alteration of ß-catenin’s apico-lateral distribution, alteration of Cx43’s apical distribution, loss/mis-localization of Scrib’s apico-lateral expression, and with decreased nuclear circularity. miR-183-5p and miR-492 over-expression triggered enhanced proliferation in cells cultured in both 2D and 3D conditions as well as enhanced invasion capacity when cultured under conditions with permissive or reduced ECM stiffness. Thus, miR-183-5p or miR-492 over-expression in the polarized S1 cells recapitulated the pretumorigenic phenotypic changes that were previously observed upon silencing Cx43 in S1 cells^29,30^.

### miR-183-5p and miR-492 act as oncomiRs

To understand the potential mechanism of action of miR-183-5p in breast cancer, IPA was used and revealed that the experimentally validated and predicted mRNA target(s) downstream of miR-183-5p in breast cells and tissues were (1) PDCD4, FOXO1 and BTRC (involved in invasion and proliferation pathways) and (2) MMP9, NFKB1, PTEN, SMAD4, TCF4, WNT2B, WNT5A and ZEB-1 (involved in epithelial polarity pathways). Cheng et al. showed that miR-183-5p expression level were significantly up-regulated in breast cancer patient samples as compared to normal adjacent tissues^43^. miR-183-5p over-expression resulted in a significant increase in cell proliferation and decrease in apoptosis in MCF-7 and MDA-MB-231 cells, which was predicted to be regulated by its downstream target and tumor suppressor, programmed cell death 4, PDCD4. Knocking down PDCD4 resulted in downregulating p21 and p27 and thus in enhancing cell proliferation and decreasing apoptosis^43^. In another study, miR‐183 was confirmed among a biomarker panel of miRNAs whose up-regulation predicted the tumor progression of lobular neoplasia from an in situ to a malignant transformation through loss of cellular polarity and acquisition of a hyperplastic phenotype^11^. This is in concordance with the increased expression of miR-183-5p that was recorded with the increased risk of cancer progression in the S1 3D culture model shown in Fig. 1f, and consistent with the reported oncogenic role of miR-183-5p in breast tumors^44^.Therefore, miR-183-5p over-expression, through acting as an oncomiR, is likely driving these pre-tumorigenic phenotypes in the breast epithelium.

Using IPA, the experimentally validated and predicted mRNA target(s) downstream of miR-492 in breast cells and tissues were (1) CD44 (invasion and proliferation pathways), (2) CCL21, HPN and GDF5 (proliferation pathways), and (3) and ETS1, LTF, RARG, RHOJ, TGFB2 and ELF5 (epithelial polarity pathways). Although, miR-492 has not been widely studied in breast cancer like miR-183-5p has, it seems to play an essential tumor-initiation role in various cancer types^45^. Shen et al. showed that miR-492 was significantly up-regulated in breast cancer tissues and cells. Its over-expression triggered proliferation and anchorage-independent growth in cells and acted as an oncomiR in breast cancer initiation through associating with cyclin D1 and c-Myc and downregulating SOX7, a tumor suppressor and a downstream target of miR-492. Therefore, the study revealed that a direct SOX7 supression is instrumental in the pretumorigenic phenotypes observed in breast cancer cells upon the over-expression of the oncomiR, miR-492^45^. miR-492 was also predicted as an early diagnostic cancer biomarker in breast, colorectal, ovarian, and pancreatic cancers as well as in hepatocellular carcinoma and retinoblastoma^46,47^. Of note, miR-492 was exclusively found up-regulated in the young Lebanese patient cohort but not the matched US population^10^, which might propose a diagnostic role during early breast cancer stages in this young patient population cohort. It also was only up-regulated in the studied Lebanese patients below the age of 40, but not in the subset of the cohort above 40 years of age^10,16^. Therefore, miR-492, through acting as an oncomiR, might be driving the pretumorigenic phenotypes and might act as an early diagnostic biomarker for breast cancer.

### Epithelial polarity is central to normal phenotype and its loss is an early lesion in tumorigenesis

miR-183-5p or miR-492 over-expression recapitulate pre-tumorigenic phenotypes seen upon Cx43 loss, however, the involvement of miR-183-5p and miR-492 with gap junctions is not yet clear. While Cx43 is not a down-stream target of miR-183-5p or miR-492, as predicted by TargetScan, miR-183-5p was found up-regulated in Cx43-KO-S1 cells as compared to S1 cells, which creates a link between Cx43 loss and miR-183-5p up-regulation. Moreover, miR-183-5p up-regulation altered the apical distribution of Cx43. On the other hand, miR-492 was not found dysregulated in Cx43-KO-S1 cells as compared to S1 cells, but its over-expression was able to alter the localization of Cx43 in S1 acini. Using TargetScan, the predicted gap junctional downstream target(s) of miR-183-5p was Cx36 and for miR-492 were Cx30.3, Cx25, and Cx31.9. Therefore, miR-183-5p or miR-492 over-expression might, in part, disrupt polarity in a gap junctional dependent manner, through the mislocalization of Cx43 from apical membrane domains, and by downregulating other downstream gap junctional and other cell junction gene targets, like cadherins, claudins, occludin, desmocollins, among others, as reported in Targetscan (data not shown).

Of note, low levels of Cx43 in the primary breast tumors at initial stages of the malignancy associate with poor prognosis^28^. Conversely, high levels of Cx43 in breast cancer patients at later tumor stages are associated with poor prognosis and correlate with enhanced tumor progression and invasion^48^. This is observed as the tumor epithelial cells increase their gap junctional intercellular communication with endothelial cells, in order to enhance intravasation and extravasation to invade and metastasize into neighboring tissues^16,17^. Therefore, Cx43 acts as a tumor suppressor during early tumorigenesis, and its loss promotes breast cancer initiation^29^, and progression^30^ and associates with poor prognosis^28^, however, Cx43 re-expression at later tumor stages enhances invasion and metastasis and correlates with poor prognosis^48^. We recently proposed Cx43/hsa_circ_0077755/miR-182 as a potential biomarker signature axis for heightened-risk of breast cancer initiation, and that this axis acts as a temporal prognostic axis along breast cancer initiation and progression^16^. miR-183-5p acts similarly to miR-182 in breast cancer, since they belong to the same miR-183 family cluster^35^. Interestingly, survival analysis revealed that miR-183-5p, when up-regulated, associates with poor prognosis in grade 1 breast tumors, however, in grade 3 breast tumors, miR-183-5p is associated with poor prognosis when downregulated. As for miR-492, it correlates with poor prognosis when up-regulated in breast tumors regardless of the tumor grade, when referencing the METABRIC breast cancer miRNA dataset^38,39^. However, in the exceptionally young Lebanese patient cohort, miR-492 was only up-regulated in the studied Lebanese patients below the age of 40, but not in the subset of the cohort above 40 years of age^10,16^. This implies that miR-183-5p is in synch with the validated Cx43/miR-182 axis; meaning when Cx43 is down (acting as a tumor suppressor) and miR-183-5p is up (acting as an oncomiR) at low tumor grades, they are associated with poor prognosis, while when Cx43 is up and miR-183-5p is down at high tumor grades, they are associated with poor prognosis. Additionally, miR-183-5p was up-regulated upon the loss of Cx43 in S1 cells. Thus miR-183-5p over-expression seems to be downstream of Cx43 loss and epithelial polarity disruption. On the contrary, miR-492 was not associated with Cx43 loss, as it was not differentially expressed in Cx43-KO-S1 compared to S1 miRNome, and was up-regulated at all grades of the malignancy in the breast cancer database, but only in the young patients below the age of 40 in the Lebanese population, suggesting that it might be acting upstream of Cx43 loss/mis-localization and epithelial polarity disruption, especially in the young Lebanese patient cohort. Therefore, epithelial Polarity/architecture is central to normal phenotype and its loss is an early lesion in tumorigenesis, whether the injury is upstream or downstream. Building on our results, future work will focus on validating downstream mRNA targets of miR-183-5p and miR-492 in the risk progression culture model and compare it to the transcriptome of the young Lebanese patient population.

## Conclusion

Ectopic expression of miR-183-5p or miR-492 in 3D cultures of non-neoplastic S1 cells resulted in cell multi-layering, formation of larger acini that lack a lumen, and in decreased nuclear circularity. Acini exhibited disrupted epithelial polarity observed through mislocalization of ß-catenin and Scrib’s apico-lateral distribution and Cx43’s apical distribution. Over-expression of miR-183-5p or miR-492 in S1 cells also enhanced proliferation in 2D and 3D cultures and triggered invasion in permissive/reduced ECM stiffness, hence recapitulating pretumorigenic phenotypes seen upon Cx43 loss^29,30^. Of note, Cx43 acts as a tumor suppressor, miR-183-5p acts as an onco-miR, and miR-492 as both an onco-miR and possibly as an early diagnostic biomarker for breast tumors. Results from this study suggest that Cx43 and miR-183 act as differential prognostic biomarker in a tumor-grade dependent manner while miR-492 acts as poor prognostic marker when up-regulated along all grades of the malignancy, and an early diagnostic biomarker for the young population cohort in this study. We propose that miR-183-5p up-regulation is downstream of Cx43 loss and epithelial polarity disruption while miR-492 up-regulation is not attributed to Cx43 loss but induces epithelial polarity disruption. Therefore, epithelial polarity/architecture is central to normal phenotype, its loss is an early lesion in tumorigenesis and a biomarker for the malignancy whether the injury is upstream (miR-492 over-expression) or downstream of it (miR-183-5p over-expression).

## Materials and methods

### Three-dimensional cell culture

The 3D cell culture was maintained as described by Naser Al Deen et al.^16^. Briefly, non-neoplastic HMT-3522 S1 (S1) human breast epithelial cells^40^ between passages 52 and 60, were routinely maintained as a monolayer on plastic (2D culture) in chemically defined serum-free H14 medium^49,50^ at 37 ˚C and 5% CO2 in a humidified incubator. H14 medium was changed every 2–3 days. For 2D cultures, cells were plated on plastic flasks at a density of 2.3 × 10^4^ cells/cm^2^. The drip method of 3D culture was used to induce the formation of acini. Cells were plated on 100% Matrigel™ (50 μl/cm^2^; BD Biosciences, 354,234) at a density of 4.2 × 10^4^ cells/cm^2^ in the presence of culture medium containing 5% Matrigel™^49,51^. The EGF was omitted from the culture medium after day 7 to allow completion of acinar differentiation (usually observed on day 8 or 9)^49^. Cx43 was down-regulated in S1 cells via retroviral delivery of shRNA, as described by Bazzoun et al.^29^.

### Total RNA isolation and quality control (QC)

Total RNA from cells in 3D culture was extracted using TRIzol reagent (Invitrogen, Carlsbad, CA, USA) following the manufacturer’s protocol and as described by Naser Al Deen et al.^16^. Spectrophotometrically by absorbance measurements at 260, 280 and 230 nm using the NanoDrop ND-1000 (Thermo Fisher Scientific, Wilmington, DE, USA) was used to assess the purity and concentration of RNA samples. OD260/OD280 ratios between 1.8 and 2.1 were considered acceptable.

### miRNA library preparation and sequencing

Triplicate samples of S1 cells and triplicates of Cx43-KO-S1 cells were submitted for small RNA-seq as described by Naser Al Deen et al.^16^. Breifly, the Purdue Genomics Facility prepared libraries using the NEXTflex Illumina Small RNA Sequencing Kit v3 (Bioo, Austin, TX) with barcoding performed using UDI primers. A total of 15 PCR cycles were performed. 2 × 50 bp reads were sequenced using the NovaSeq6000. Only one of each read pair was used for analyses, given the short size of miRNAs. Library preparation protocols were modified for the use of unique dual indexes, in order to circumvent index hopping on the Illumina NovaSeq 6000. The RNA quality was checked using an Agilent Nano RNA Chip prior to library preparation.

### Heatmap of miRNAs from the breast epithelial 3D cell culture dodel

Adapters were removed from the left reads of each sample and trimming was performed using cutadapt version 1.13^52^. A total of 4 bases were removed off of either side of the reads and bases at the end of reads with base quality Phred scores lower than 20 were removed. Trimmed reads were aligned using Bowtie version 1.3.1^53^ with the –best flag, with one mismatch permitted, and a seed length of 18 to the human reference genome assembly GRCh38. During alignment, the maximum permitted total of quality values at all mismatched read positions permitted throughout the entire alignment is 80 and all valid alignments per read were reported. Reads were counted to features using FeatureCounts from the Subread package, v. 1.6.1^54^. The resulting count matrix was normalized and a differential expression analysis performed using DESeq2^55^. The calculated p-values were corrected for multiple testing using the Benjamini–Hochberg method. miRNAs were determined to be differentially expressed at an alpha of 0.05 and a fold-change ≥ 2. In the heatmap shown in Fig. 1a, the log2 of 1 + the normalized counts was scaled by row. Rows were clustered using hierarchical clustering and were annotated with the direction (up- or down-regulation) of the associated circRNAs in Cx43-KO-S1 samples versus S1 control samples.

### Functional enrichment of mRNAs associated with the differentially expressed miRNAs

A functional enrichment analysis was performed in Ingenuity Pathway Analysis (IPA®, QIAGEN Redwood City, www.qiagen.com/ingenuity) of predicted target mRNAs (predicted by TargetScan) for the differentially expressed miRNAs in Cx43-KO-S1 cells as compared to S1 cells as described by Naser Al Deen et al.^16^.

### miRNA expression by quantitative real time-polymerase chain reaction

Reverse transcription of ten nanograms of the total RNA was performed using the TaqMan® MicroRNA Reverse Transcription Kit (Applied Biosystems, USA) according to the manufacturer’s instructions. Small nuclear RNA RNU6B (control sequence: CGCAAGGATGACACGCAAATTCGTGAAGCGTTCCATATTTTT), hsa-miR-183-5p (mature miRNA sequence: UAUGGCACUGGUAGAAUUCACU), hsa-miR-182-5p (mature miRNA sequence: UUUGGCAAUGGUAGAACUCACACU) and hsa-miR-492 (mature miRNA sequence: AGGACCUGCGGGACAAGAUUCUU) primers and probes (Assay ID: 001093, 002269, 002334, 001039, respectively) were purchased and used for RT-qPCR validation as part of the TaqMan® microRNA Assays Kit (Applied Biosystems, USA) with validated efficiency.Briefly, small nuclear RNA RNU6B, miR-183-5p and miR-492 primers and probes were purchased as part of the TaqMan® microRNA Assays Kit (Applied Biosystems, USA) with validated efficiency. cDNA synthesis was carried out in a multiplex reaction set up whereby two miRNA primers (miR-183-5p and miR-492) were used in each reaction with the endogenous control, RNU6B. RT-qPCR was performed using BioRad CFX96 Real Time System, C1000 Thermal Cycler (Germany) as described by Naser Al Deen et al.^16^. The results were analyzed in Prism GraphPad (version 9.2.0) based on unpaired t-test with Welch’s correction.

### Lentiviral infection for delivery of mature miRNAs into S1 cells

The pLenti-III-miR-GPF tagged vectors from ABM (Canada) were used to generate lenti-miR-183-5p, lenti-miR-492, and lenti-miR-control (empty vector) with kanamycin resistance for over-expression in S1 cells in bacterial culture and puromycin resistance to infect S1 cells and generate stable miR-183-S1 and miR-492-S1 as described by Naser Al Deen and Talhouk^56^. Briefly, plasmids were streaked in an agar plate and grown overnight in a humidified incubator at 37 °C, a single colony was selected and amplified in agar broth overnight in an incubator shaker, and then purified using Qiagen Midi-Prep Kit as per the company’s protocol. Briefly, lentiviral vectors (10 μg) containing pLenti-III-miR-GPF tagged vectors for each of the mature transcript of miR-183, miR-492 or the miR-Control (empty vector) were transfected into the amphoteric 293 T packaging cells using Invitrogen™ Lipofectamine™ 2000 (Themo Fisher Scinetific, USA) according to manufacturer’s protocol (80 μL). and a 2nd Generation Packaging Mix (10 μg) in 1 mL of serum-free, antibiotic-free medium for each 10 cm dish and incubated at 37 °C for 5–8 h, then 0.65 mL FBS was added to the 10 cm dish and incubated overnight at 37 °C. The transfection media was replaced with complete media and incubated overnight at 37 °C. The first viral harvest in the supernatant was collected at 3000 rpm for 15 min at 4 °C, filtered and stored at 4 °C for the next day. Complete media was added again to the cells and a second harvest was collected and filtered the next day and added to the first harvest that was kept at 4 °C. The pooled harvest was aliquoted and stored at – 80 °C until use. The viral titer of the first harvest is approximately 106 IU/mL. Puromycin drug selection killing curve showed that concentration of 0.5 μg/ul of Puromycin was optimal for selecting the infected cells as per manufacturer’s protocol (ABM, Canada). Multiplicity of Infection, MOI was calculated as: Product Titer (IU/ml) × Virus Volume (ml)/Total Cell Number, and 3 MOI was used for the S1 cells, as recommended by ABM for breast cell lines. For infection, filtered lentiviral supernatants were applied to monolayers of S1 cells on day 4 in culture. Cells were incubated with hexadimethrine bromide (Polybrene; 8 μg/mL; Sigma, St. Louis, MO, USA) for 8 h. The infection medium was removed, and cells were incubated in regular H14 medium for 24 h. Infection was repeated two additional times on days 5, and 6 of culture, and selection with puromycin dihydrochloride (0.5 ug/ul; Gibco™, USA, A-1113803) was started 72 h after the last infection (day 9). miRNA-infected cells (miR-183-S1, miR-492-S1 and miR-Control-S1) cells were maintained, propagated, and plated similarly to S1 cells, but the H14 medium was supplemented with puromycin for selection. The stability of the over-expression was regularly assessed in different cell passages with fluorescence microscopy and RT-qPCR throughout this study.

### Immunofluorescence

S1, miR-183-S1, miR-492-S1 and miR-Control-S1cells were plated on 4-well chamber slides (3D) and were stained by immunofluorescence on day 11 as described by Fostok et al.^30^. Briefly, cells were washed with 1 × PBS and permeabilized with 0.5% peroxide and carbonyl-free Triton X-100 in cytoskeleton buffer (100 mM NaCl, 300 mM sucrose, 10 mM PIPES, pH 6.8, 5 mM MgCl2, 1 mM pefabloc, 10 μg/mL aprotinin, 250 μM NaF). Cells were washed twice with cytoskeleton buffer and fixed in 4% formaldehyde. Cells were subsequently washed three times with 50 mM glycine in 1 × PBS and blocked. Primary antibodies used were mouse monoclonal β-catenin (200 μg/mL; Santa Cruz Biotechnology, Dallas, TX, USA, sc-7963), mouse monoclonal Scrib (200 μg/mL; Santa Cruz Biotechnology, Dallas, TX, USA, sc-55543) and mouse monoclonal Connexin 43 (200 μg/mL; Santa Cruz Biotechnology, Dallas, TX, USA, sc-271837) all at dilutions (1:200). Secondary antibody conjugated to Alexa Fluor 568 (red), goat anti-mouse (Invitrogen, Waltham, MA, USA, A-11004) was used according to the manufacturer's recommended dilutions (1:1000). Nuclei were counterstained with 0.5 μg/mL Hoechst 33342, and cells were mounted in ProLong® Gold antifade reagent, dried overnight and sealed. The slides were then examined and imaged with a laser scanning confocal microscope (Zeiss, LSM710). A minimum of 100 acini were analyzed per group.

### Diameter measurements, lumen assembly and apico-lateral polarity markers scoring

The fixed slides were examined with an upright fluorescent microscope (Leica, DF7000 T) using a 40 × oil immersion objective to measure the diameter of 3D cultures, assess lumen assembly, and score for the apicolateral distribution of β-catenin. A minimum of 100 acini were analyzed per group. The size of acini was measured manually by recording the diameter of each acinus or nodule using LAS X software. Lumen assembly was scored visually. Apicolateral distribution of β-catenin was scored visually from acini having proper apico-lateral distribution. At least 100 acini were included in the measurements and scoring.

### Nuclear circulatory scoring

ImageJ was used to calculate the change in nuclear morphometry using the DAPI images of the triplicates of each cell line, and measuring circularity (0–1) in at least 150 nuclei per replicate and for at least 10 acini/aggregates per replicate.

### Trypan blue exclusion method

S1, miR-183-S1, miR-492-S1 and miR-Control-S1 cells were plated in 12-well tissue culture plates in 2D and in Cells were plated on 100% Matrigel™ (50 μl/cm^2^; BD Biosciences, 354,234) at a density of 4.2 × 10^4^ cells/cm^2^ in the presence of culture medium containing 5% Matrigel™ using the drip method of 3D culture to induce the formation of acini, as described in “Three-dimensional cell culture” methods section above. The medium was removed, and the cells were subsequently trypsinized and collected. Cells were then diluted in trypan blue at 1:1 ratio (vol/vol) and counted using a hemocytometer from triplicates on days 5, 9 and 13 in 2D cultures and on days 7, 11 and 13 in 3D culture.

### Transwell cell invasion assay

Six-well cell culture inserts of 8 μm pore size were coated with 400 μL of Matrigel™ (1:5 dilution) and incubated at 37◦C for 4 h to solidify as described by Fostok et al.^30^. Briefly, 3 × 10^5^ total S1, miR-183-S1, miR-492-S1 or miR-Control-S1cells were plated on the inserts in DMEM:F-12 supplemented with 1% fetal bovine serum (FBS; Sigma, F-9665). Below the insert, DMEM:F-12 with 10% FBS was added. Cells were incubated for 72 h and were then fixed using 4% formaldehyde in 1 × PBS for 20 min at room temperature. The nuclei of invading cells were stained with 1 μg/mL Hoechst 33342 (Molecular Probes, H3570) in 1 × PBS for 10 min at room temperature. The inserts were then cut, mounted on a microscope slide in ProLong® Gold antifade reagent (Molecular Probes, P36930), left to dry overnight and sealed. The inserts were then examined with a fluorescence microscope and brightfield since miR-183-S1, miR-492-S1 and miR-Control-S1cells were infected with the pLenti-III-miR-GPF tagged vectors, and the number of invading cells was counted and reported as fold change (Supplementary information S1).

## Supplementary Information

Supplementary Information.

## Data Availability

The raw and processed datasets generated from microarray and sequencing analyzed during the current study are available on the Geobrowser (Accession number: GSE196062).
