# Supplementary material for: Over-expression of miR-183-5p or miR-492 triggers invasion and proliferation and loss of polarity in non-neoplastic breast epithelium
Source: Sci Rep. 2022 Dec 20;12:21974. doi: 10.1038/s41598-022-25663-8 (PMC9768134; doi:10.1038/s41598-022-25663-8)
Supplement: Supplementary file 1 — Supplementary Information. [file 41598_2022_25663_MOESM1_ESM.pdf]

Supplementary Figures:

S1

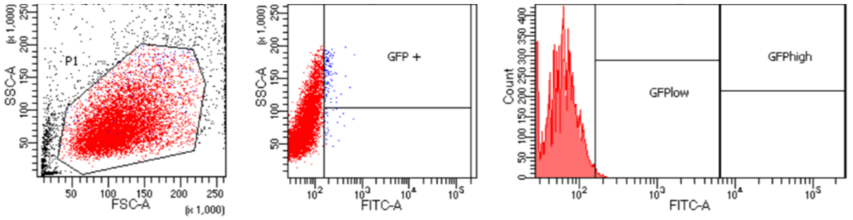

Tube: US

Population	#Events	%Parent	%Total
All Events	10,000	####	100.0
P1	8,227	82.3	82.3
GFP +	114	1.4	1.1
GFPlow	111	1.3	1.1
GFPhigh	0	0.0	0.0

miR-183-S1

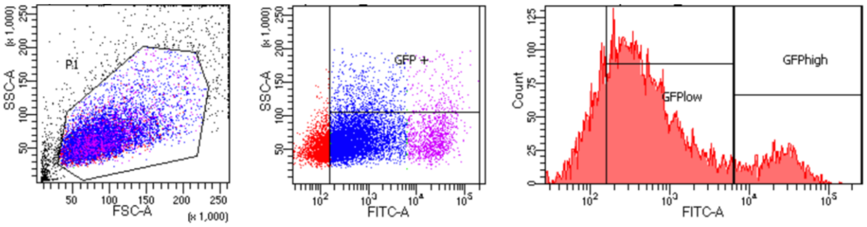

Tube: miR183

Population	#Events	%Parent	%Total
All Events	10,000	####	100.0
P1	8,907	89.1	89.1
GFP +	7,114	79.9	71.1
GFPlow	5,914	66.4	59.1
GFPhigh	1,176	13.2	11.8

miR-492-S1

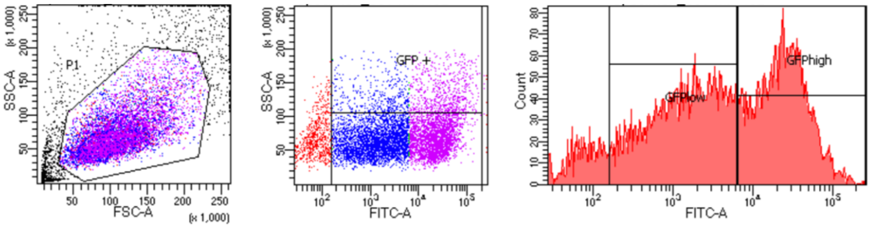

Tube: miR492

Population	#Events	%Parent	%Total
All Events	10,000	####	100.0
P1	7,763	77.6	77.6
GFP +	7,078	91.2	70.8
GFPlow	3,680	47.4	36.8
GFPhigh	3,360	43.3	33.6

Supplementary Figure 1. Quantification of stable over-expression of miR-183-5p and miR-492 in non-neoplastic S1 cells through pLenti-III-miR-GPF tagged vectors using fluorescence-activated cell sorting (FACS). After all cell lines were stably established, they were cultured in 2D for 11 days, and FACS was performed to identify the GFP+ parent percent, as highlighted in a red box.
